# Topical Treatments in Atopic Dermatitis: An Expansive Review

**DOI:** 10.3390/jcm13082185

**Published:** 2024-04-10

**Authors:** Michelle Lazar, Aurore D. Zhang, Neelam A. Vashi

**Affiliations:** Department of Dermatology, Boston University School of Medicine, 609 Albany St., J502, Boston, MA 02118, USA

**Keywords:** atopic dermatitis, inflammatory skin conditions, topical therapy, steroids, quality of life, over-the-counter treatments, cost of treatment

## Abstract

Atopic dermatitis (AD) is a common inflammatory skin condition found worldwide. It impacts patient quality of life (QoL) and is thought to arise as an inflammatory response to epidermal barrier dysfunction and hypersensitivity. AD can lead to large out-of-pocket costs and increased healthcare expenses over a lifetime. An analysis of all randomized control trials conducted since 1990 on topical therapies for AD were reviewed, including 207 trials in the final analysis. It was found that an average of 226 patients were enrolled over 2.43 arms. Common topical treatments included corticosteroids, calcineurin inhibitors, JAK inhibitors, and phosphodiesterase inhibitors. The most utilized tools to identify treatment efficacy were the EASI, IGA, SCORAD, and PGA. There was a paucity of data on trials that evaluated efficacy, QoL, and cost of treatment simultaneously. This review highlights the need for comprehensive trials that evaluate multiple aspects of treatment, including financial cost and QoL impact, to ensure each patient has the best treatment modality for the management of their AD.

## 1. Introduction

Atopic dermatitis (AD) is one of the most common inflammatory skin conditions. With an estimated lifetime prevalence of 3.0% to 17.7%, it is a condition that many individuals will be diagnosed with over the course of their life [1]. AD commonly first presents in the early years of life and often remits spontaneously by adolescence or adulthood [1]. As a result, not only the severity of the AD but also the age of the patient must be considered prior to starting treatment. Many patients try to manage their AD with over-the-counter (OTC) medications and remedies [2]. Therefore, a large portion of healthcare spending for AD is paid for out-of-pocket by patients. A recent survey study of patients with AD in the United States found that the median out-of-pocket expense was $600, with 41.9% of patients reporting spending over $1000 annually for the treatment and management of their AD [2]. This can lead to a large fiscal burden on patients and frustration when treatments fail to resolve or improve their AD.

The exact pathophysiology behind atopic dermatitis is not fully understood, but it is likely due to a complex interplay of epidermal barrier dysfunction, alterations in the classical immune response, skin hypersensitivity, and environmental factors [3]. These features all coincide to create an environment where the skin is uniquely fragile and prone to xerosis and superinfection. Additionally, there is early evidence that genetic factors may play a role in the pathogenesis of AD, wherein genetically sensitive individuals with mutations in the filaggrin gene are exposed to environmental factors that lead to the development of AD [4]. This explains why atopic dermatitis is often clustered in families and helps to explain the high comorbidity with other so-called “atopic” conditions such as asthma and allergic rhinitis. Some research has posited that effective management of AD may reduce the development of other atopic diseases [5]. Therefore, effectively managing AD is important not only for patient quality of life (QoL) but may also help decrease overall morbidity.

Atopic dermatitis can present at any age, though it is most prevalent in children [1]. Typically, atopic dermatitis presents as dry, scaly erythematous plaques and patches. These lesions can lead to skin shedding, lichenification, and intense pruritus. AD is commonly categorized using a variety of clinical scales. One of these is the EASI, which was developed in 1998 [6]. In the EASI, the body is divided into four regions which are then scored based on erythema, edema, excoriation, lichenification, and percent body surface area. Another score commonly used is the Investigator’s Global Assessment (IGA). The IGA utilizes a simple scoring system from 0 to 4 wherein scorers rate a lesion as clear, almost clear, mild, moderate, or severe [7]. A third commonly used metric is the SCORAD, which was developed in 1993 in response to complaints with other industry models [8]. Today, it exists on a myriad of websites as an easy to use calculator to quickly score the progression of AD over time. These scores can be used to measure disease severity and therefore guide treatment plans. 

The severity of AD largely dictates the treatments available. Mild AD can be treated with OTC emollients and lotions [9]. However, when OTC agents do not offer relief, topical steroids are utilized as a mainstay in the treatment of atopic dermatitis [10]. Steroids have been posited to have their effect by decreasing the abnormal inflammatory response seen in AD, which helps reduce lesion severity and allows the skin barrier to heal [10]. Similarly, tacrolimus is an immunosuppressive topical agent that also alters the local microenvironment of AD to help promote skin healing. Other options for treating AD include phototherapy, but further research needs to be conducted to understand the long term effects of this therapy and potential sequalae [11]. 

Due the highly prevalent nature of AD and the ever-changing landscape, we sought to better characterize the research conducted thus far on topical treatments for AD. The aim of this review was to summarize topical treatment options for AD, identify commonly used metrics to score AD, summarize the impact of AD on QoL, and highlight gaps in current evidence and the literature to ascertain what ought to be studied next.

## 2. Materials and Methods

**Figure 1:**
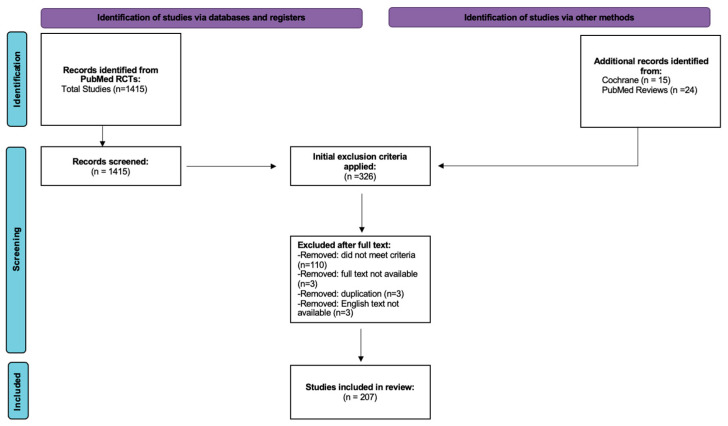
PRISMA-based flow diagram depicting inclusion and exclusion criteria. In total, 207 unique studies were included in this analysis.

A flow diagram of search criteria can be found in Figure 1. A PubMed search was conducted with the phrase “atopic dermatitis” and included all randomized control trials from 1990 to 2024. This resulted in 1415 total papers. PubMed was then queried again for all systematic reviews and meta-analyses with the phrase “atopic dermatitis topical” in the last decade. This yielded 133 articles, 15 of which were cross-referenced against the initial search to identify additional articles, from which an additional 24 papers were added. Following this, all Cochrane articles published were reviewed for the phrase “atopic dermatitis”, yielding 15 additional papers. The final net result was 1454 papers. 

Following this, studies were excluded if they did not meet the following inclusion criteria: randomized clinical trials, total number of patients enrolled was 20 or greater, and published at any point after 1990. Studies were excluded if they utilized non-topical treatments in any arm of the study, focused on combined disease states, had no clear diagnostic criteria of AD defined, or were a phase I pharmacokinetic or safety trial. Split design studies were excluded, as were studies that focused on AD with active infection. Trials that had not reported data or whose full text could not be located were also excluded. Additionally, any study that did not have the full text available in English was excluded. All trials that had multiple papers published on the same trial group were combined and analyzed as one study. The result was 207 unique studies published since 1990 that utilized topical treatments in the management of AD [12,13,14,15,16,17,18,19,20,21,22,23,24,25,26,27,28,29,30,31,32,33,34,35,36,37,38,39,40,41,42,43,44,45,46,47,48,49,50,51,52,53,54,55,56,57,58,59,60,61,62,63,64,65,66,67,68,69,70,71,72,73,74,75,76,77,78,79,80,81,82,83,84,85,86,87,88,89,90,91,92,93,94,95,96,97,98,99,100,101,102,103,104,105,106,107,108,109,110,111,112,113,114,115,116,117,118,119,120,121,122,123,124,125,126,127,128,129,130,131,132,133,134,135,136,137,138,139,140,141,142,143,144,145,146,147,148,149,150,151,152,153,154,155,156,157,158,159,160,161,162,163,164,165,166,167,168,169,170,171,172,173,174,175,176,177,178,179,180,181,182,183,184,185,186,187,188,189,190,191,192,193,194,195,196,197,198,199,200,201,202,203,204,205,206,207,208,209,210,211,212,213,214,215,216,217,218,219,220,221]. 

Each entry was inputted into a shared Google Sheet and PubMed IDs were utilized to identify papers when available; otherwise, the DOI or full citation was used. Papers were reviewed by the authors M.L. and A.D.Z. The extracted data included total number of participants, total number of participants after attrition, age of patients enrolled, treatment arms, number of patients enrolled per arm, duration of treatment, measurement tool for efficacy, statistical significance of efficacy, rates of flares and relapses, statistical significance of flares and relapses, QoL metrics, significance of QoL data, statistical significance of patient preference, cost of affiliated treatment, statistical significance of cost, and other features. 

## 3. Results

### 3.1. Demographic Data of Studies

Two-hundred and seven unique studies were analyzed from 1990 to 2024 and demographic data were extracted (Table 1). The average study enrolled 226 patients, with a minimum of 20 patients enrolled and a maximum of 2439 patients enrolled. One-hundred and nineteen trials reported on attrition or the total number of patients who completed full treatment course. After accounting for attrition, there was an average of 207 patients enrolled across trials. Only 13 studies reported no attrition from their study, while the largest percentage attrition was 79.07%. The average attrition rate for all studies was 15.49%, or 13.90% when the three highest attrition rates were removed. One-hundred and ninety-three studies reported on the average age of patients enrolled in their trial. Of these trials, 154 enrolled pediatric patients, defined as individuals under the age of 18, in their trial. The youngest participant to be enrolled was 2 weeks old and the oldest was 87 years old. Sixty-one trials included both adult and pediatric patients. There were an average of 2.43 arms to each trial, with a minimum of two arms and a maximum of 8 arms. Two-hundred and three trials reported the duration of treatment. The shortest duration of treatment was 7 days and the longest was 5 years. On average, studies were carried out for 82.98 days. Most commonly, trials were conducted for 28 days. 

### 3.2. Commonly Utilized Treatments

There were a total of 83 unique treatments analyzed over all studies. The most common treatments were placebo treatments, consisting of OTC lotions, creams, gels, emollients, and oils—present in 154 arms. Tacrolimus was the next most studied agent, present in 53 study arms. Pimecrolimus was identified in 34 arms. Fluticasone was studied in 24 study arms. Hydrocortisone was studied in 22 study arms. Branded moisturizing lotions were studied in 11 study arms. Of the treatments studied, there were four major groups of agents (Table 2). The largest subcategory was corticosteroids, consisting of 96 arms. The commonly studied corticosteroids included fluticasone (24), hydrocortisone (22), triamcinolone (7), fluocinonide (6), clobetasol (5), and mometaonse (5). Calcineurin inhibitors were the next most commonly studied, consisting of 87 arms focused on tacrolimus and pimecrolimus. Janus Kinase (JAK) inhibitors were the third most commonly studied category of treatments, contributing to 22 trial arms. JAK inhibitors included by name included ruxolitinib, brepocitinib, delgocitinib, and tofacitinib. Phosphodiesterase inhibitors were studied across 14 trial arms and included E6005, difamilast, OPA-15406, and roflumilast. 

Twenty-two trials directly compared the efficacy of topical steroids to calcneinurin inhibitors. Calcineurin inhibitors were found to be more effective than topical steroids in eight trials, and significantly more effective in reducing disease severity in seven trials (*p* < 0.05). Five trials found that steroids either in combination with calcineurin inhibitors or steroids alone reduced disease severity to a greater extent. Nine trials were non-inferiority trials that found no difference in the efficacy of calcineurin inhibitors as compared to standard of care topical corticosteroids. Three trials compared JAK inhibitors to topical steroids or calcineurin inhibitors. These trials found that the effect of the JAK inhibitors demonstrated a dose-dependent response. Five trials directly compared the efficacy of different topical steroids. Of these, one trial found that desonide 0.05% was more efficacious than 1% hydrocortisone. One found no difference in efficacy between 1% hydrocortisone and 0.1% betamethasone valerate. A third trial found no difference in efficacy between 0.05% fluticasone propionate and 0.05% clobetasone butyrate. Finally, two trials found a statistically significant difference in AD clearance favoring mometasone furoate over hydrocortisone (*p* < 0.05). Six trials utilizing phosphodiesterase inhibitors showed significant improvement in AD severity as compared to placebo (*p* < 0.05).

### 3.3. Statistical Significance 

**Figure 2:**
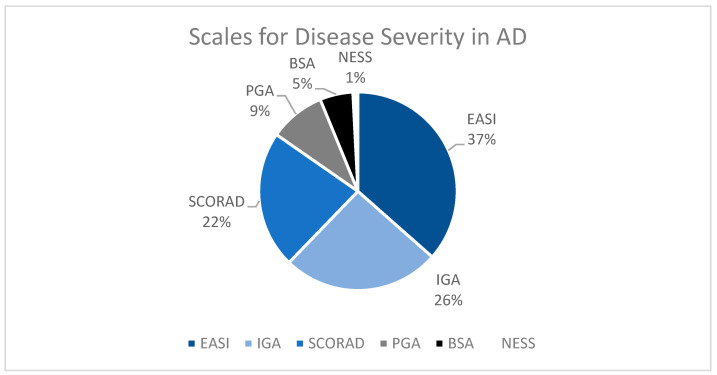
Graphical representation of commonly used metrics for measurement of disease severity and treatment progression in AD.

The primary efficacy of treatment was defined by most studies as score changes on measurements of disease severity (Figure 2). In total, 88 studies utilized the Eczema Area and Severity Index (EASI), 62 the Investigator’s Global Assessment (IGA), 54 the SCORing Atopic Dermatitis (SCORAD), 22 the Physician’s Global Assessment (PGA), 13 Body Surface Area (BSA), and 2 the Nottingham Eczema Severity Score (NESS). All but 15 studies included statistical analyses of their primary endpoint. Of studies that reported significance, 36 reported no difference between treatments or lack of efficacy of treatment. One-hundred and thirty-three studies reported significant outcomes for their primary endpoint (*p* < 0.05).

Thirty-one studies commented on relapse rates and disease recurrence. This was measured in disease-free time, time to flare, time between flares, relapse rate, severity of flare, and the quantity of steroids needed during flares. Only 4 studies of all 207 analyzed studies included any data regarding cost and potential burden for patients and conducted no statistical analysis on these metrics. 

Fifty studies included data on quality of life (QoL). Commonly used metrics included the dermatology life quality index (DLQI), Children’s DLQI (CDLQI), Infant DLQI (IDLQI), Skindex-16, and the 36-Item Short Form (SF-36). Fourteen studies utilized the DLQI, twelve the CDLQI, nine the IDLQI, three the Skindex-16, and two studies the SF-36. Of trials utilizing QoL metrics, eleven reported no effect of treatment on QoL. Treatments that were found to have no effect on QoL were topical OTC agents, urea-based lotions, tetraconazole, pimecrolimus, and pimecrolimus with topical steroids. Conversely, 24 trials found that one or more treatments led to an improvement in QoL. The treatments found to have a statistically significant effect on QoL were: ruxolitinib, tacrolimus, topical OTC lotions, crisaborole, difamilast, fluticasone, hydrocortisone, delgocitinib, and jaungo. A summary of the significant results as reported by the trials can be found in Table 3. 

## 4. Discussion

Atopic dermatitis (AD) is a common condition that presents across all age groups [1]. Based on Global Burden of Disease (GBD) estimates, AD ranks 15th among all non-fatal diseases and first among all skin conditions regarding the impact on disability-adjusted life years (DALYs) [222]. Thousands of papers on AD have been published trying to better characterize AD and identify the best management practices. However, not all studies are equal in the quality of data they provide. When characterizing studies, it is important to not only consider the findings but what is omitted as well. 

One important metric of a study is the attrition rate. An attrition rate is the number of individuals who left a study, either due to failure of treatment compliance, loss to follow up, unexpected adverse event, death, or unknown reason. Research has indicated that attrition rates across medical research can be as high as 20% or more, even in leading medical journals [223]. This greatly impacts the findings that can be postulated from study data, as it is difficult to identify why patients are not completing treatment or were lost to follow up. Attrition rates can lead to loss of power in studies, meaning that the claims the authors postulate may not be numerically supported. While many trials aim to over-enroll to ensure that they are sufficiently powered even after accounting for estimated attrition, this too can lead to problems as many trials require extensions to fulfill enrollment criteria [224]. Therefore, authors and journals alike must consider standard attrition rates for their disease of choice prior to designing studies, to ensure they are powered enough to make statistical claims. Per our review, we identified an average attrition rate of 15.49% across all studies who readily made such data available. The average enrollment size for AD trials was 226; therefore, if investigators wish to have a final sample size of 226, they ought to aim to enroll roughly 270 patients. Researchers also ought to ensure that all treatments are as equal as possible to prevent unequal attrition rates between arms, which can further skew data. 

Per our analysis, many trials focused on pediatric populations and adult populations separately. Identifying a proper study population is vital to create findings that can be generalized to the target group. This review highlighted that, on average, trials on topical treatments for AD tended to have 2.43 arms. Most studies included in this review compared two products to better understand which treatments had the best efficacy. Additionally, many trials aimed to prove non-inferiority between industry standards and novel treatments rather than improvements in efficacy between treatments. 

The most studied topical treatments for atopic dermatitis were corticosteroids, calcineurin inhibitors, JAK inhibitors, and phosphodiesterase inhibitors. One of the first articles utilizing hydrocortisone in the treatment of AD was published in 1952, and topical steroids have continued to be a mainstay of treatment since [225]. While highly effective in the management of AD, topical steroids come with a long list of potential adverse events such as skin atrophy, telangiectasia, and even severe withdrawal when topical steroids are stopped [226]. Therefore, it is of no surprise that many patients are concerned about the use of steroids in their treatment. A recent study found that regardless of how risks are framed, many patients are hesitant to utilize topical steroids in the management of AD, even with the large amount of data backing this treatment modality [227]. As a result, new strategies to manage AD have been developed, one of which are calcineurin inhibitors. Calcineurin inhibitors reduce interleukin-2 (IL-2) production, leading to less T cell activation and decreased immune response [228]. Most commonly, topical calcineurin inhibitors are either 0.03% tacrolimus, 0.1% tacrolimus, or 1.0% pimecrolimus [228]. One of the benefits to calcineurin inhibitors is that they do not lead to skin atrophy even with long term use and can help relieve the pruritus associated with AD [229]. Our analysis indicated that calcineurin inhibitors were found to be more effective than topical steroids in eight trials, and significantly more effective in reducing disease severity in seven trials (*p* < 0.05).

Janus kinase inhibitors (JAK inhibitors) are a newer modality of treatment for AD that inhibit JAK signaling and thus blunt the cytokine signaling normally associated with inflammation [230]. However, not all JAK inhibitors are the same as they have differences in selectivity of inhibition, degree of inhibition, and safety profiles [230]. Our analysis found that three trials found that JAK inhibitors demonstrated a dose-dependent response in improving disease severity (*p* < 0.05).

Finally, phosphodiesterase inhibitors are also commonly used in the management of AD. By blocking phosphodiesterase, these agents can stop the degradation of cyclic adenosine monophosphate (cAMP), which reduces overall inflammation [231]. Six trials utilizing phosphodiesterase inhibitors showed significant improvement in AD severity as compared to placebo (*p* < 0.05). Other systematic reviews on the subject have found that phosphodiesterase inhibitors are safe and effective in the management of mild to moderate AD, and therefore are frequently utilized in its treatment [6]. Due to the large amount of treatment modalities in AD, trials conducted on AD should seek to identify the most efficacious agent in each class and between classes. 

There are a variety of treatments that can be utilized in the management of AD. Therefore, it is important to have standardized tools with which providers can identify disease severity and change over time to quantify treatment success or the need for additional agents. One of the most utilized tools in our analysis was the Eczema Area and Severity Index (EASI), which was first developed in 1998 [6]. The EASI has been validated and is frequently used in AD research as well as clinical practice. However, the EASI is not the only metric used in AD research. Other commonly used tools in our analysis were the Investigator’s Global Assessment (IGA), SCORing Atopic Dermatitis (SCORAD), Physician’s Global Assessment (PGA), percentage of body surface area (BSA), and the Nottingham Eczema Severity Scale (NESS). While each tool has pros and cons, the variety of tools can make it difficult to compare results across trials. Therefore, investigators ought to ensure that they use a well-recognized metric when adding to the body of literature on AD. 

The trials utilized disease clearance as a common endpoint per our analysis as measured by the scales listed above. In our analysis, there was a paucity of trials that reported non-significant findings for their primary endpoint. Publication bias, or the tendency for journals to not publish findings that are not statistically significant, is well studied [232]. However, negative findings are still findings and can greatly benefit the scientific community. Knowing what treatments do not work is often just as important as knowing the treatments that do. There continues to be a strong publication bias in the field of AD that may be leading to duplication of studies and wasted resources. Journals ought to consider publishing negative findings as well if they contribute meaningfully to the field. 

Atopic dermatitis can be a chronic condition and thus has a large impact on patient quality of life (QoL), especially if a patient’s disease is more severe. There are many metrics that can be utilized to identify the impact AD has on QoL. In the field of dermatology, commonly used metrics include the dermatology life quality index (DLQI), Children’s DLQI (CDLQI), Infant DLQI (IDLQI), Skindex-16, and the 36-Item Short Form (SF-36). Some trials reported on patient pruritus and improvement with treatment; however, we found that only 50 studies commented on the impact of AD on patient quality of life (QoL). While there are a myriad of papers that focus solely on the impact of AD on QoL, most studies do not comment on disease progression and treatment efficacy while including QoL. The separation of these two topics can hide important data, such as patient preference and patient-perceived disease burden. Trials conducted on AD ought to include QoL data alongside efficacy data to ensure that treatments are not only efficacious but lead to impacts on a patient’s QoL as well.

Finally, our analysis indicates that there is a large paucity of trials that comment on both the efficacy and cost of treatments in AD, present in only 4 of the 207 papers analyzed. This is concerning as it is known that healthcare costs are on average higher for patients with AD than for matched controls, largely driven by the need for a variety of topical treatments [233]. More research needs to be conducted on the cost of AD treatments to ensure patients can afford the treatments being offered. Treatment is only effective if accessible to patients; therefore, having data regarding cost is important when counseling patients on the treatments available. 

One of the strengths of this review is the large time span covered, the comprehensive search criteria, and the large number of studies reviewed. Additionally, the structured nature of the analysis allowed for a thorough investigation of each study and its strengths and weakness. One of the limitations of this study is that only topical treatments were analyzed, excluding all systemic therapies that are often utilized in more severe disease states. Additionally, no statistical analysis could be conducted between studies due to the lack of comparable arms between trials. Another limitation of this study is that by excluding non-English papers and research not indexed on PubMed, there are likely missing data from international trials. Future research ought to examine studies in comparison to identify if statistical significance can be identified between trials to compare the efficacy of treatments not directly compared. 

## 5. Conclusions and Future Directions

AD is one of the most common skin conditions. We found that most trials conducted on topical treatments in AD had 2.43 arms and 226 enrolled patients. The treatments commonly studied included corticosteroids, calcineurin inhibitors, JAK inhibitors, and phosphodiesterase inhibitors. The most utilized tools to identify treatment efficacy were the EASI, IGA, SCORAD, and PGA. There is a continued need for trials that evaluate the efficacy, QoL, and cost of treatments simultaneously to ensure that the best treatment modality for each patient can be appropriately identified. 

## Figures and Tables

**Table 1:** Demographic data of studies reviewed.

Category	Average	Minimum	Maximum
Total (*n*)	226	20	2439
Total Completed (*n*)	207	-	-
Attrition Rate	15.49%	0%	79.07%
Age	-	2 weeks	87 years
Treatment Arms	2.43	2	8
Duration (days)	82.98	7	1825

**Table 2:** Subgroups most commonly studied.

Corticosteroids	Calcineruin Inhibitors	JAK Inhibitors	Phosphodiesterase Inhibitors
96 arms	87 arms	22 arms	14 arms

**Table 3:** Overall findings.

Metrics	Number of Trials
Analysis of AD severity	171 trials
Decreased AD severity with treatment, significant	156 trials
Analysis of AD relapse rate	31 trials
Decreased AD relapse rate, significant	23 trials
QoL analysis	50 trials
Improved QoL with treatment, significant	24 trials

## Data Availability

The raw data supporting the conclusions of this article can be made available by the authors on request.
